# Back to the Future! Understanding old mechanisms and discovering new insights via ADSP initiatives

**DOI:** 10.1002/alz.093159

**Published:** 2025-01-03

**Authors:** William S. Bush, Anthony J. Griswold

**Affiliations:** ^1^ Department of Population and Quantitative Health Sciences, Institute for Computational Biology, Case Western Reserve University, Cleveland, OH USA; ^2^ Cleveland Institute for Computational Biology, Department of Population and Quantitative Health Sciences, Case Western Reserve University, Cleveland, OH USA; ^3^ John P. Hussman Institute for Human Genomics, University of Miami Miller School of Medicine, Miami, FL, USA, Miami, FL USA

## Abstract

The Alzheimer’s Disease Sequencing Project (ADSP) has used whole genome sequencing, computational approaches, and epidemiological and statistical expertise to accelerate understanding of the fundamental mechanisms of Alzheimer disease and related disorders (ADRD). Going forward, the ADSP will continue growing to over 110,000 participants to further dissect the risk for AD. To move towards novel insights, the ADSP is adopting new approaches while leveraging the rich existing data. New technologies such as long‐read sequencing will reveal complex structural variants in diverse genetic ancestries and enable discovery of novel variants for further exploration. The ADSP Functional Genomics Consortium will expand their work in the generation and integration of multiomic data to better elucidate underlying disease mechanisms thereby enhancing therapeutic target discovery. Harnessing the ADSP data resource will allow examination of genetic heterogeneity by ancestry, identifying both shared and ancestry‐specific genetic variants that modulate AD risk, encompassing variants of all types using a variety of new statistical approaches and integration with large AD‐specific multiomics resources such as through xQTL projects. The phenotypic heterogeneity of AD will also be examined through the collection and harmonization of emerging AD fluid and imaging biomarkers and cognitive testing through the efforts of the ADSP Phenotype Harmonization Consortium. The ADSP AI/ML consortium will develop new analysis approaches for many of these tasks and will construct comprehensive risk models for AD by incorporating genetics, endophenotypes, biomarkers, and social determinants of health. Finally, as we enter an era of new Alzheimer’s therapeutics, the ADSP is poised to uncover genetic predictors of treatment response and adverse events as these are adopted in the general population, enabling personalized medicine approaches for maximizing benefit and minimizing harm. Through expanding sample diversity, integrating diverse datasets, applying cutting‐edge technologies, and forging global collaborative networks, the Alzheimer’s Disease Sequencing Project aims to achieve an unprecedented understanding of Alzheimer’s disease genetics—ultimately translating genetic insights into life‐changing treatments and prevention strategies worldwide.

